# Chasing Chances in a Changing Sea

**DOI:** 10.3390/md20050311

**Published:** 2022-05-03

**Authors:** Ernesto Mollo

**Affiliations:** Institute of Biomolecular Chemistry, National Research Council of Italy, 80078 Pozzuoli, Italy; emollo@icb.cnr.it

Bioactive marine natural products (BMNPs) of interest for applications as drugs, antimicrobials, cosmetics, nutraceuticals, or antifoulants, are often present in traces in producer organisms and often occur in threatened or endangered species, or in organisms playing key ecological roles. Under such conditions, the commercial-scale extraction and isolation of BMNPs is unrealistic or could have a huge negative impact on the environment and biodiversity. Solving this problem calls for either the development of challenging synthetic strategies to replicate in the laboratory the most promising BMNPs, or the development of aquaculture strategies for those species that can be farmed. These issues, however, do not apply to BMNPs that can be obtained from marine invasive species (MIS), which are dramatically compromising the integrity of marine ecosystems, challenging the conservation of biodiversity on a global scale. Abundant and undesired biomaterials from MIS are having such a large impact on marine ecosystems that their exploitation to obtain high added-value products is emerging as a sustainable and highly desirable form of biomass valorization [1,2,3,4,5,6]. In this view, research programs in marine natural product chemistry and drug discovery could stimulate the harvesting of MIS, offering valuable options for reducing their impact on marine ecosystems towards knowledge-based management of marine biological invasions. It is worth considering that we have barely scratched the surface of the vast library of BMNPs that can be obtained from MIS, and many important questions remain to be assessed in the identification and characterization of the so-called “alien biomolecules” [2] in defining the mechanism of their biological activity, and in realistically evaluating their possible use in industrial applications.

The following are some examples of studies carried out on species that have invaded the Mediterranean Sea, which is particularly affected by biological invasions. Crude extracts from one of the most threatening MIS in the Mediterranean, the seagrass *Halophila stipulacea*, have been indicated as possible sources of BMNPs of interest to combat obesity and biofouling [7], while the bioactive components of the plant extracts remain to be identified. Much remains to be investigated also regarding the activity of purified BMNPs from invasive red algae of the genus *Asparagopsis*, whose extracts have already been evaluated for their antioxidant, antibacterial, antifungal, antiviral, antifouling, cytotoxic, antimethanogenic and enzyme-inhibitory properties [8]. Among benthic invertebrates, the calcareous sponge *Paraleucilla magna*, commonly occurring as an invasive fouler in Mediterranean mussel farms and marinas, could represent an important source of metabolites of interest for the development of novel environmentally friendly antifouling paints, as suggested by a preparatory study carried out by testing crude ethanolic extract from the sponge [9]. The extracts from the upside-down jellyfish *Cassiopea andromeda* showed considerable antioxidant activity, paving the way for further studies envisaging a sustainable exploitation of this invasive species as a source of BMNPs with nutritional, nutraceutical and/or pharmaceutical properties [10].

Although those mentioned are just a few examples from a growing literature, they give an idea of how much work remains to be done in the identification of the bioactive ingredients that can be obtained from MIS. This Special Issue of *Marine Drugs* entitled “Marine Invasive Species and Their Bioactive Metabolites” aims to fill some of these gaps, giving priority for publication to submissions that might promote eco-innovation by helping to turn relevant ecological threats into opportunities for sustainable development of the sea-based economy (Figure 1). 

## Figures and Tables

**Figure 1:**
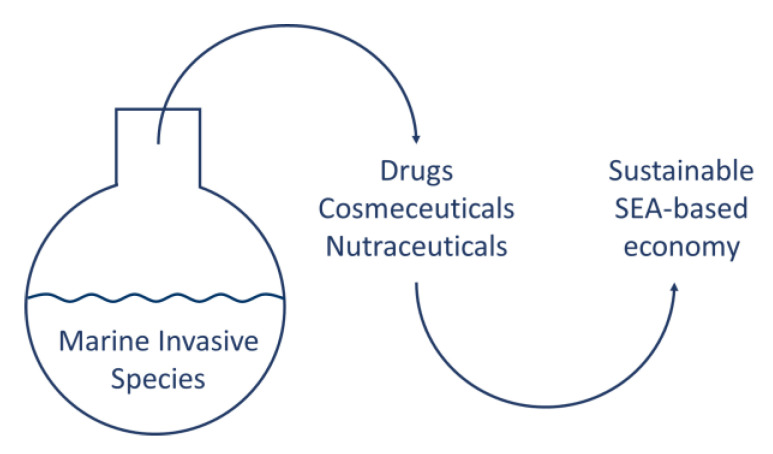
Conceptual scheme.
